# Total Cholesterol and the Risk of Parkinson's Disease: A Review for Some New Findings

**DOI:** 10.4061/2010/836962

**Published:** 2009-11-10

**Authors:** Gang Hu

**Affiliations:** Chronic Disease Epidemiology Laboratory, Population Science, Pennington Biomedical Research Center, 6400 Perkins Road, Baton Rouge, LA 70808, USA

## Abstract

The studies on the association between serum cholesterol level and the risks of neurodegenerative diseases risk are debated. Some prospective studies have found that high serum cholesterol may increase the risks of dementia/Alzheimer's disease and ischemic stroke. However, other studies have found no association or a decreased risk of hemorrhagic stroke with increasing levels of serum total cholesterol. Little is known about the association between serum total cholesterol or a history of hypercholesterolemia and Parkinson's disease (PD) risk. Only a few case-control studies and four prospective epidemiological studies have examined this association, but the results are inconsistent. An inverse association between serum total cholesterol and the risk of PD has been found in one prospective study; however, no significant association is reported in the case-control studies and other two prospective studies. Recently, one large prospective study from Finland suggests that high total cholesterol at baseline is associated with an increased risk of PD. Further studies, especially large clinical trials, are needed.

## 1. Parkinson's Disease

Parkinson's disease (PD) is the second most common neurodegenerative disease of the elderly [1]. PD is a debilitating disorder manifested by bradykinesia, resting tremor, muscular rigidity, gait disturbances, and postural reflex impairment [1]. The underlying pathologic lesion is the loss of the pigmented neurons of the substantia nigra, and selected brain stem dopaminergic cell groups. However, the causes of PD are not well known. The loss of substantia nigra neurons results in depletion of the neurotransmitter dopamine in these areas. There is evidence that genetic factors play a key role in development of PD [2, 3]. Studies of twins have provided strong evidence for an important role of environmental factors in the etiology of typical PD [2]. The development of PD involves an interaction between genes and environmental factors [2]. Thus, its prevention is likely to be at least partly possible. In order to determine interventions that would prevent or delay the onset of PD, modifiable risk factors for the disease have to be identified first. These risk factors could then be used as targets for intervention, and also population-based health education and intervention programs could be developed. Because the process leading to the clinical manifestation of PD takes decades, long-term prospective studies are necessary to identify its environmental risk factors.

## 2. Serum Total Cholesterol

Cholesterol is a lipid found in the cell membranes of all animal tissues and is transported in the blood plasma of all animals. Most of the cholesterol is synthesized by the body and some have dietary origin. Cholesterol is more abundant in tissues which either synthesize more or have more abundant densely-packed membranes, for example, the liver, spinal cord, and brain. It plays a central role in many biochemical processes, such as the composition of cell membranes and the synthesis of steroid hormones.

There is good evidence that high serum total cholesterol level increases coronary heart disease risk in both middle- and old-aged people and in people at all blood pressure levels [4]. However, the association between serum cholesterol level and several neurodegenerative disease risks has been debated. Some prospective studies have found that high serum cholesterol may increase the risks of dementia/Alzheimer's disease [5, 6] and ischemic stroke [4]. Other studies have found no association [7] or a decreased risk of hemorrhagic stroke with increasing levels of serum total cholesterol [4]. In addition, little is known about the association between serum total cholesterol or a history of hypercholesterolemia and PD risk.

## 3. Serum Total Cholesterol and the Risk of PD

Until now, only four case-control studies [8–11] and four prospective epidemiological studies [12, 14–16] have examined the association between serum total cholesterol or a history of hypercholesterolemia and the risk of PD, but the results are inconsistent (Table 1). 

### 3.1. Results from Case-Control Studies

In two small case-control studies, serum total cholesterol did not differ significantly between PD patients and controls [8, 9] (Table 1). In one large retrospective case-control study including 178 newly diagnosed consecutive idiopathic PD patients and 533 age- and sex-matched controls, the investigators did not find any association between serum total cholesterol and the occurrence of idiopathic PD [10]. In one recent case-control study including 124 PD cases and 112 controls, serum total cholesterol did not differ significantly between PD patients and controls; however, use of cholesterol-lowering drugs in this population was associated with a lower occurrence of PD, the odd ratio was 0.36 (95% CI 0.19–0.68) after adjusting for age, gender, and smoking status [11]. Moreover, it indicated that lower concentrations of low-density lipoprotein (LDL) cholesterol were associated with higher occurrence of PD [11]; however, serum high-density lipoprotein (HDL) cholesterol did not differ significantly between PD patients and controls [11]. These case-control studies indicate that serum total cholesterol did not differ significantly between PD patients and controls.

### 3.2. Results from Prospective Studies

The prospective association of serum total cholesterol or a history of hypercholesterolemia with PD risk has been assessed recently [12, 14–16]. In the Honolulu Heart Program including 8006 Japanese-American men in Hawaii identified 58 PD cases during a 26-year followup [12], the original aim of this study was to assess the association of cigarette smoking with PD risk. The age adjusted relative risk of PD was 0.73 (95% CI 0.43–1.24) [12]. In an updated analysis from the Honolulu Heart Program, an inverse association between LDL cholesterol and PD risk was presented among 3233 subjects [13]. In the Rotterdam Study documenting 87 cases of PD among 6465 Netherlanders during an average 9.4 years of followup [14], higher levels of serum total cholesterol were associated with a significantly decreased risk of PD (hazard ratio 0.77, 95% CI 0.64–0.94 per 1.0 mmol/L increase in serum total cholesterol). In the further analyses, the age- and sex-adjusted hazard ratios of incident PD across quartiles of serum total cholesterol were 1.0, 0.82 (95% CI 0.48–1.41), 0.55 (95% CI 0.30–1.02), and 0.55 (95% CI 0.30–1.04), respectively [14]. With the pooled data from Harvard, the Health Professionals Follow-up Study involved 50 833 men identified 266 PD cases, and the Nurses' Health Study involved 121 046 women identified 264 PD cases during followup [15]. The history of physician-diagnosed high cholesterol was collected by questionnaire. Age- and smoking-adjusted relative risk of PD was not associated with the baseline history of high cholesterol (relative risk 0.98, 95% CI 0.82–1.19) [15].

We evaluated prospectively the association between serum total cholesterol and the risk of incident PD among 24 773 Finnish men and 26 153 women of 25 to 74 years of age without a history of PD and stroke at baseline [16]. Serum total cholesterol was measured at baseline survey. During a mean followup period of 18.1 years, 321 men and 304 women developed incident PD. The multivariable-adjusted (age, study year, body mass index, systolic blood pressure, education, leisure time physical activity, smoking, alcohol drinking, coffee and tea drinking, cholesterol-lowering agent use, and the history of diabetes) hazard ratios of incident PD at different levels of total cholesterol (<5, 5–5.9, 6–6.9, and ≥7 mmol/L) were 1.00, 1.33, 1.53, and 1.84 (*P* for trend = .035) in men, 1.00, 1.55, 1.57, and 1.86 (*P* for trend = .113) in women, and 1.00, 1.42, 1.56, and 1.86 (*P* for trend = .002) in men and women combined (adjusted also for sex) (Table 2). When total cholesterol was examined as a continuous variable, multivariable-adjusted hazard ratios of PD for each 1 mmol/l increase in total cholesterol were 1.10 (95% CI 1.01–1.21) for men, 1.07 (95% CI 0.98–1.17) for women, and 1.09 (95% CI 1.02–1.16) for men and women combined (Table 1). After exclusion of participants who used cholesterol-lowering agents (*n* = 371), sex- and multivariate-adjusted hazard ratios of PD at different levels of total cholesterol (<5, 5–5.9, 6–6.9, and ≥7 mmol/l) were 1, 1.42 (95% CI 0.99–2.02), 1.54 (95% CI 1.09–2.18), and 1.85 (95% CI 1.30–2.61) (*P* for trend = .002) [16].

In the multivariate analyses, the increased risk of PD associated with increasing levels of total cholesterol was present both in subjects aged 25–44 years (*P* for trend = .025) and 45–54 years (*P* for trend = .011) at baseline, and in never smokers (*P* for trend = .061) and smokers (*P* for trend = .044), however, no association was found among subjects aged 55 years or more at baseline (*P* for trend = .98) (Table 2) [16].

To our knowledge, this Finnish study is the first large prospective study to find out that high total cholesterol may increase PD risk. However, no significant association is reported by the case-control studies and other two prospective studies, and an inverse association has been found in one prospective study. Several reasons for the inconsistency of these studies can be considered. First, potential bias in the case of PD could be substantial in these case-control studies [8–11]. Second, an older age at baseline in the Rotterdam Study (over 55 years) [14] could also be considered since the association between total cholesterol and the risk of PD in Finnish elder study samples (55–74 years) was not significant either [16]. Third, cholesterol levels seem to decline with the development of many chronic diseases including neurodegenerative disorders like Alzheimer's disease [17] before the clinical manifestation of the disease. Previous results from the Finnish study indicate that high mid-life serum total cholesterol is a risk factor for subsequent dementia/Alzheimer's disease [6], and that decreased serum total cholesterol after mid-life may reflect ongoing disease processes and may represent a risk marker for dementia/Alzheimer's disease [17]. Further studies are needed to confirm whether this is also true for PD. Fourth, the history of high cholesterol was assessed by questionnaire and no available data measured serum total cholesterol in the Health Professionals Follow-up Study and the Nurses' Health Study making the data vulnerable to various biases [15]. Fifth, few PD cases in these two prospective studies may limit the statistical power [12, 14]. Sixth, since ascertainment of the PD cases in Finnish study was based on the National Social Insurance Institution's Register on special reimbursement for PD drugs, people with high total cholesterol level are more prone to other diseases and therefore are likely to be in contact with the health care system more often than people with normal total cholesterol. This could not be easily justified and may result in an ascertainment bias [16]. However, we think this is less likely for PD, because PD symptoms are clearly defined, and likely to bring a subject to the attention of the medical system [16].

## 4. Biological Mechanisms

Even though the Finnish study finds an increased PD risk associated with high levels of total cholesterol, the mechanisms of this association are poorly understood. Several putative mechanisms may be proposed. This association may however not be completely unexpected given that brain is the most cholesterol rich organ in the whole body; disturbances in cholesterol homeostasis may therefore influence the structure, function, and maintenance of neuronal cell membranes and synapses [18]. The aggregation of alfa-synuclein is believed to play a critical role in the pathogenesis of PD. Interestingly, recent data indicate that change in cholesterol composition using cholesterol-lowering agents reduces the levels of alfa-synuclein in vitro [19]. Alfa-synuclein is also known to influence brain lipid metabolism [20]. The epidemiologic studies have shown that high serum cholesterol is associated with an increased risk of dementia/Alzheimer's disease [5, 6], and statins may delay the progression of Alzheimer's disease and PD [11, 21]. For the inverse association between serum total cholesterol and the PD risk in the Rotterdam Study [14], they proposed that the strong correlation between levels of serum cholesterol and the antioxidant coenzyme Q10 may support for of oxidative stress in the pathogenesis of Parkinson's disease.

It could also be hypothesized that high total cholesterol might increase the risk of PD partly through excess body weight. The results from the Honolulu Heart Program and our study have demonstrated that excess weight is associated with an elevated PD risk [22, 23]. In the Finnish study, there was a positive association between BMI and serum total cholesterol [16]. However, the positive association of total cholesterol with PD risk in Finnish study was independent of baseline BMI [16]. A decreased PD risk associated with an increased leisure-time physical activity level has been found among men in the Health Professionals Follow-up Study but not among women in the Nurses' Health Study [24]. Recently, The Finnish study also found that a history of diabetes was associated with an increased risk of PD [25]. However, taking into account these possible effects by adjusting for baseline habits of leisure-time physical activity, coffee consumption [26], history of diabetes, and other risk factors [27] including baseline systolic blood pressure did not change the positive association of total cholesterol and the risk of PD in the Finnish Study [16].

Results from case-control studies have suggested an association between PD and dietary factors, such as total, saturated, and animal fat intake and energy intake [28, 29], but the results from prospective studies are conflicting. In Honolulu Heart Program among Japanese-American men, no association between diet and the risk of PD was seen [23]. In the Health Professionals Follow-up Study and the Nurses' Health Study, higher baseline intakes of animal fat and saturated fat tended to increase the risk of PD in men (*P* = .1 for trend) but not in women [30]. It could be hypothesized that high serum total cholesterol might increase the risk of PD partly through dietary factors.

## 5. Conclusions

The studies on the association between serum total cholesterol and a history of hypercholesterolemia and the risk of PD are few the results are inconsistent. A decreased risk of PD with higher serum levels of total cholesterol has been found in one prospective study; however, no significant association is reported in the case-control studies and other two prospective studies. The result from one recent large Finnish prospective study has shown that high total cholesterol at baseline is associated with an increased risk of PD. This increased risk of PD associated with increasing levels of serum total cholesterol was present both in subjects aged 25–44 years and 45–54 years at baseline, and no association was found among subjects aged 55 years or more at baseline. Since most above studies only had baseline measurement of total cholesterol and changes in the levels of total cholesterol were not available during followup, further studies, especially large clinical trials, are needed in the next several years. It would be important to find out mechanisms behind the association between serum total cholesterol and PD risk.

## Figures and Tables

**Table 1:** Prospective studies of total cholesterol and the risk of Parkinson's disease.

Lead author, year [ref.]	Samples or controls/cases	Age (years)	Sex	Mean values of cholesterol (case : control) (mmol/L)	*P*-value for difference	Relative risk (95% CI)	Adjusted for confounding factors
Case-control studies							

Teunisse et al., 2003 [8]	61/46	68/70		6.41 : 5.59	n.s.		
							
Sohmiya et al., 2004 [9]	29/36	59/63		4.65 : 4.20	n.s.		
							
Sciglian et al., 2006 [10]	533/178	60/58	Both			0.89 (0.68–1.15)	Age and sex
							
Huang et al., 2007 [11]	112/124	66/68	Both	5.19 : 4.96	n.s.		

Cohort studies							

Grandinetti et al., 1994 [12]	8006/58	53	Male			High cholesterol (yes/no): 0.73 (0.43–1.24)	Age
							
Huang et al., 2008 [13]	3222/41	77	Male			—	—
							
De Lau et al., 2006 [14]	3811/41	69	Female			Per 1.0 mmol/L increase:	Age and sex
	2654/46	68	Male			0.77 (0.64–0.94)	
							
Simon et al., 2007 [15]	121 046/264	42	Female			High cholesterol (yes/no):	Age, smoking, hypertension, diabetes
	50 833/266	54	Male			0.98 (0.82–1.19)	
							
						Per 1.0 mmol/L increase:	Age, sex, body mass index, systolic blood pressure, education, leisure time physical activity, smoking, coffee, tea, and alcohol consumption, cholesterol-lowering agent use, and history of diabetes
Hu et al., 2008 [16]	26 153/304	44	Female			1.09 (1.02–1.16) for both	
	24 773/321	44	Male			1.10 (1.01–1.21) for men	
						1.07 (0.98–1.17) for women	

n.s.: not significant.

**Table 2:** Hazard ratio (HR) of Parkinson disease according to different levels of total cholesterol among various subpopulations [16]*.

HR (95% CI)	Total cholesterol (mmol/L)				*P* for trend
	<5	5–5.9	6–6.9	≥7.0	
Total samples	1.00	1.42 (1.00–2.03)	1.56 (1.10–2.21)	1.86 (1.31–2.63)	.002
Gender					
Men	1.00	1.33 (0.81–2.16)	1.53 (0.96–2.46)	1.84 (1.14–2.95)	.035
Women	1.00	1.55 (0.92–2.61)	1.57 (0.93–2.64)	1.86 (1.11–3.13)	.113
Age (years)					
25–44	1.00	1.90 (1.05–3.45)	1.79 (0.97–3.30)	2.54 (1.37–4.70)	.025
45–54	1.00	1.34 (0.67–2.67)	1.65 (0.85–3.20)	2.20 (1.14–4.24)	.011
55–74	1.00	1.00 (0.56–1.79)	1.07 (0.61–1.88)	1.03 (0.59–1.81)	.98
Smoking					
Never	1.00	1.34 (0.85–2.12)	1.57 (1.01–2.45)	1.74 (1.11–2.71)	.061
Ever or current	1.00	1.58 (0.89–2.79)	1.55 (0.88–2.72)	2.05 (1.18–3.59)	.044

*Adjusted for age, sex (exception in gender analyses), study year, body mass index, systolic blood pressure, education, leisure-time physical activity, smoking (except in smoking status analysis), coffee, tea, and alcohol consumption, cholesterol-lowering agent use, and history of diabetes.

## References

[1] Lang AE, Lozano AM (1998). Parkinson’s disease. First of two parts. *The New England Journal of Medicine*.

[2] Tanner CM, Ottman R, Goldman SM (1999). Parkinson disease in twins: an etiologic study. *Journal of the American Medical Association*.

[3] Sveinbjornsdottir S, Hicks AA, Jonsson T (2000). Familial aggregation of Parkinson’s disease in Iceland. *The New England Journal of Medicine*.

[4] Lewington S, Whitlock G, Clarke R (2007). Blood cholesterol and vascular mortality by age, sex, and blood pressure: a meta-analysis of individual data from 61 prospective studies with 55,000 vascular deaths. *The Lancet*.

[5] Notkola IL, Sulkava R, Pekkanen J (1998). Serum total cholesterol, apolipoprotein E epsilon 4 allele, and Alzheimer’s disease. *Neuroepidemiology*.

[6] Kivipelto M, Helkala EL, Laakso MP (2002). Apolipoprotein E epsilon4 allele, elevated midlife total cholesterol level, and high midlife systolic blood pressure are independent risk factors for late-life Alzheimer disease. *Annals of Internal Medicine*.

[7] Suh I, Jee SH, Kim HC, Nam CM, Kim IS, Appel LJ (2001). Low serum cholesterol and haemorrhagic stroke in men: Korea Medical Insurance Corporation Study. *The Lancet*.

[8] Teunissen CE, Lutjohann D, Von Bergmann K (2003). Combination of serum markers related to several mechanisms in Alzheimer’s disease. *Neurobiology of Aging*.

[9] Sohmiya M, Tanaka M, Tak NW (2004). Redox status of plasma coenzyme Q10 indicates elevated systemic oxidative stress in Parkinson’s disease. *Journal of the Neurological Sciences*.

[10] Scigliano G, Musicco M, Soliveri P, Piccolo I, Ronchetti G, Girotti F (2006). Reduced risk factors for vascular disorders in Parkinson disease patients: a case-control study. *Stroke*.

[11] Huang X, Chen H, Miller WC (2007). Lower low-density lipoprotein cholesterol levels are associated with Parkinson’s disease. *Movement Disorders*.

[12] Grandinetti A, Morens DM, Reed D, Maceachern D (1994). Prospective study of cigarette smoking and the risk of developing idiopathic Parkinson’s disease. *American Journal of Epidemiology*.

[13] Huang X, Abbott RD, Petrovitch H, Mailman RB, Ross GW (2008). Low LDL cholesterol and increased risk of Parkinson’s disease: prospective results from Honolulu-Asia Aging Study. *Movement Disorders*.

[14] De Lau LM, Koudstaal PJ, Hofman A, Breteler MM (2006). Serum cholesterol levels and the risk of Parkinson’s disease. *American Journal of Epidemiology*.

[15] Simon KC, Chen H, Schwarzschild M, Ascherio A (2007). Hypertension, hypercholesterolemia, diabetes, and risk of Parkinson disease. *Neurology*.

[16] Hu G, Antikainen R, Jousilahti P, Kivipelto M, Tuomilehto J (2008). Total cholesterol and the risk of Parkinson disease. *Neurology*.

[17] Solomon A, Kareholt I, Ngandu T (2007). Serum cholesterol changes after midlife and late-life cognition: twenty-one-year follow-up study. *Neurology*.

[18] Reiss AB, Siller KA, Rahman MM, Chan ES, Ghiso J, De Leon MJ (2004). Cholesterol in neurologic disorders of the elderly: stroke and Alzheimer’s disease. *Neurobiology of Aging*.

[19] Bar-On P, Rockenstein E, Adame A, Ho G, Hashimoto M, Masliah E (2006). Effects of the cholesterol-lowering compound methyl-*β*-cyclodextrin in models of *α*-synucleinopathy. *Journal of Neurochemistry*.

[20] Barcelo-Coblijn G, Golovko MY, Weinhofer I, Berger J, Murphy EJ (2007). Brain neutral lipids mass is increased in *α*-synuclein gene-ablated mice. *Journal of Neurochemistry*.

[21] Jick H, Zornberg GL, Jick SS, Seshadri S, Drachman DA (2000). Statins and the risk of dementia. *The Lancet*.

[22] Hu G, Jousilahti P, Nissinen A, Antikainen R, Kivipelto M, Tuomilehto J (2006). Body mass index and the risk of Parkinson disease. *Neurology*.

[23] Abbott RD, Ross GW, White LR (2002). Midlife adiposity and the future risk of Parkinson’s disease. *Neurology*.

[24] Chen H, Zhang SM, Schwarzschild MA, Hernan MA, Ascherio A (2005). Physical activity and the risk of Parkinson disease. *Neurology*.

[25] Hu G, Jousilahti P, Bidel S, Antikainen R, Tuomilehto J (2007). Type 2 diabetes and the risk of Parkinson’s disease. *Diabetes Care*.

[26] Hu G, Bidel S, Jousilahti P, Antikainen R, Tuomilehto J (2007). Coffee and tea consumption and the risk of Parkinson’s disease. *Movement Disorders*.

[27] Hernan MA, Takkouche B, Caamano-Isorna F, Gestal-Otero JJ (2002). A meta-analysis of coffee drinking, cigarette smoking, and the risk of Parkinson’s disease. *Annals of Neurology*.

[28] Logroscino G, Marder K, Cote L, Tang MX, Shea S, Mayeux R (1996). Dietary lipids and antioxidants in Parkinson’s disease: a population-based, case-control study. *Annals of Neurology*.

[29] Johnson CC, Gorell JM, Rybicki BA, Sanders K, Peterson EL (1999). Adult nutrient intake as a risk factor for Parkinson’s disease. *International Journal of Epidemiology*.

[30] Chen H, Zhang SM, Hernan MA, Willett WC, Ascherio A (2003). Dietary intakes of fat and risk of Parkinson’s disease. *American Journal of Epidemiology*.

